# OA03 Trauma secondary to a horse riding accident as a potential accelerating factor in inflammatory axial sponyloarthropathy: a case report and literature review

**DOI:** 10.1093/rap/rkad070.003

**Published:** 2023-09-27

**Authors:** Rida Zainab, Fahd Ali Khan, Adnan Akram Bhatti, Kate Chapman

**Affiliations:** James Cook University Hospital, Middlesbrough, United Kingdom; James Cook University Hospital, Middlesbrough, United Kingdom; James Cook University Hospital, Middlesbrough, United Kingdom; James Cook University Hospital, Middlesbrough, United Kingdom

## Abstract

**Introduction:**

Ankylosing spondylitis (AS) is a chronic inflammatory rheumatic disease characterised by axial involvement and sacroiliitis. The etiology of AS remains elusive, with genetic and environmental factors implicated in disease development and progression. This case report aims to investigate the potential association between trauma and the acceleration of AS in individuals positive for HLA-B27.

Furthermore one purpose of this case-report is to ascertain any link between horse riding accidents in jockeys or professionals undertaking horse riding as a career choice. Is there a correlation between trauma and development of inflammatory axial spondyloarthropathy subsequently or not.

**Case description:**

A 24-year-old female patient with a medical history of eczema and Graves' disease, currently undergoing propylthiouracil treatment, was referred to the rheumatology clinic due to persistent back pain for the past six to seven years following a horse riding accident. The patient had previously received physiotherapy and found relief with naproxen and co-codamol. The pain primarily affects her lower spine and radiates down her legs. While movement provides some relief, lying down exacerbates the pain. The patient experiences stiffness for approximately 30 minutes in the morning but does not exhibit peripheral joint symptoms. Exercise helps alleviate her symptoms.

The patient reported a family history of psoriasis but denied any history of uveitis, inflammatory bowel disease, weight loss, night sweats, heartblock or achilles tendinitis. She also gave a history of joint hyper mobility with reduced flexion at the spine over the years.

Blood tests revealed normal levels of ANA, CCP, ESR, rheumatoid factor, B12, HbA1c, and thyroid function. Ferritin levels were 30.2, and HLA B27 was positive.

Joint examination did not reveal any signs of synovitis. The patient exhibited good range of motion in the spine. SLR's were positive on both sides and no tenderness over spine or sacroiliac joints. Systemic Examination was normal. BASDAI score was 4. Occiput to wall distance of 0 centimeters. Beighton score 4/9.

Subsequently MRI whole spine/SI Joints were organised and results indicated a central disc protrusion at the L4-L5 level leading to critical spinal stenosis and likely impinging on the transitioning L5 and S1 nerve roots. Additionally, a disc protrusion at the L5-S1 which was impinging on the left S1 nerve root. **Bilateral sacroiliitis was also observed.**

Patient was treated with NSAIDs and physiotherapy with good results with a view for a subsequent MRI, and escalation to biologics if symptoms were to worsen.

**Discussion:**

The presented case raises the question of whether trauma accelerates the progression of ankylosing spondylitis (AS) in individuals who are positive for HLA-B27. Although this specific case involves a 24-year-old female patient with a history of back pain following a horse riding accident, additional research and studies would be required to draw definitive conclusions regarding the relationship between trauma and the development or acceleration of AS in HLA-B27 positive individuals.

The question this case report poses and there is little in terms of research and case reports present, whether the involvement of the lady in question in a horse riding accident unmasked her ankylosing spondylitis given her HLA B27 positivity and the presence of sacroilitis on MRI scans.

Literature review revealed a potential role of trauma accelerating the progression of ankylosing spondylitis in individuals who are also positive for HLA B-27, in addition in some cases it lead to detection and unmasking of chronic back pain which was inflammatory in nature rather than mechanical or a combination of both.

This was revealed by the horse riding accident given the nature of the pain which had been constant and worsening which lead to a suspicion of a possible inflammatory component and hence further investigations were done.

In conclusion this case report highlights the importance of a possible correlation between AS in HLA B27 positive individuals and raises the question whether trauma secondary to sports injuries like horseback riding accidents in our patients case, accelerates the progression of AS in individuals who are positive for HLA-B27.

**Key learning points:**

This case report highlights the potential role of trauma following a sports injury as a contributing factor in the acceleration of AS in individuals positive for HLA-B27.

Further investigations, including prospective studies, are warranted to elucidate the complex interplay between trauma, genetic predisposition, and disease progression in AS.

The purpose of this case report is to remind sports medicine physicians of the prevalence of rheumatologic diseases in general and ankylosing spondylitis in particular and of the various ways in which spondyloarthropathies may present in athletes/sports personnel.

Increased suspicion may lead to earlier diagnosis and treatment, potentially reducing illness severity and duration and improving the performance of athletes with this condition.

